# Persistence in GLP-1–Based Therapy Among Adults with Obesity and Type 2 Diabetes: A Narrative Review of Definitions, Real-World Outcomes, and Determinants

**DOI:** 10.1007/s11892-026-01639-0

**Published:** 2026-08-15

**Authors:** Tzu Wang, Olayinka O. Shiyanbola

**Affiliations:** Department of Clinical Pharmacy, College of Pharmacy, University of Michigan, Ann Arbor, MI 48104 USA

**Keywords:** GLP-1-based therapies, Type 2 diabetes, Obesity, Medication persistence, Treatment discontinuation

## Abstract

**Purpose of Review:**

Persistence with glucagon-like peptide-1 (GLP-1)–based therapies is important for sustained weight loss and glycemic control in adults with coexisting obesity and type 2 diabetes. This narrative review examined how persistence has been defined and measured, real-world patterns of continuation and discontinuation, factors associated with persistence, reasons for discontinuation, and supportive strategies in this population.

**Recent Findings:**

Seven studies met the inclusion criteria, with follow-up ranging from 6 months to 2 years. Persistence generally declined over time and often fell below 60% by 12 to 24 months, although estimates varied by refill-gap definitions and analytic approaches. Discontinuation reached 64.1% at 2 years in one large cohort, and treatment interruption with reinitiation was common. Persistence varied by age, income, gastrointestinal adverse events, weight reduction, body mass index–related measures, GLP-1 agent, formulation, and dosing schedule. Only two studies reported patient-level reasons for discontinuation, most commonly adverse effects, and none described structured behavioral or supportive strategies.

**Summary:**

Persistence with GLP-1–based therapies among adults with obesity and type 2 diabetes is variable and often declines within the first one to two years. More consistent definitions, closer attention to patient experiences, and routine-care support strategies are needed to improve long-term continuation.

**Supplementary Information:**

The online version contains supplementary material available at 10.1007/s11892-026-01639-0.

## Introduction

Obesity and type 2 diabetes frequently coexist and together represent a major global health burden. Approximately 589 million adults worldwide are living with diabetes, and a substantial proportion of individuals with type 2 diabetes are overweight or obese [1, 2]. Excess body weight is a key contributor to the development and progression of type 2 diabetes, and individuals with both conditions face increased cardiometabolic risk, greater treatment complexity, and higher rates of complications compared with either condition alone [3–6]. As a result, adults with both obesity and type 2 diabetes often require long-term treatment strategies that address both glycemic control and weight management, making sustained treatment over time a central challenge in clinical care [7, 8].

Recent clinical guidelines emphasize obesity management as a core component of diabetes care. The 2026 American Diabetes Association (ADA) Standards of Care recommend early use of weight-focused pharmacologic therapies, including glucagon-like peptide-1 receptor agonists (GLP-1 RAs) and dual glucose-dependent insulinotropic polypeptide (GIP)/GLP-1 receptor agonists, for adults with type 2 diabetes and obesity [7, 9]. These therapies have demonstrated substantial benefits in clinical trials and are intended for long-term use as part of weight-centric diabetes management [7, 9–12]. Accordingly, GLP-1–based therapies have become central to the management of adults with obesity and type 2 diabetes [7, 11, 13]. Alongside pharmacologic treatment, current guidelines also emphasize the importance of behavioral and supportive strategies, including patient education and ongoing counseling, as part of comprehensive diabetes management, which may support long-term treatment use [14].

Despite their intended long-term use, real-world evidence suggests that many patients do not remain on GLP-1–based therapies over time [15, 16]. Treatment discontinuation has been associated with weight regain and loss of metabolic benefits, highlighting the importance of sustained therapy [17–20]. Weight regain after therapy discontinuation may occur more rapidly than after behavioral weight management programs, further underscoring the importance of sustained treatment among adults with obesity and type 2 diabetes [17, 19, 21]. However, maintaining long-term use for patients may be challenging due to factors such as gastrointestinal adverse effects, treatment burden, medication cost, and patient expectations regarding treatment outcomes [13, 15, 16, 22, 23]. These challenges may be amplified in settings where structured behavioral or supportive care, such as guidance during dose titration or counseling on expected treatment trajectories, is limited [13, 14].

In this context, treatment persistence refers to the duration of time from treatment initiation to discontinuation and reflects continued treatment over time [24]. Existing evidence on persistence with GLP-1–based therapies in adults with both obesity and type 2 diabetes remains limited, and variation in definitions and measurement approaches complicates comparisons across studies [5, 25–32]. Prior research has largely focused on treatment initiation or short-term outcomes, such as weight loss and glycemic control, with less emphasis on long-term treatment continuation in this dual-condition population [16, 23, 25, 27, 28, 33]. In addition, factors underlying treatment discontinuation, including patient experiences during treatment, expectations regarding outcomes, and barriers related to cost or treatment burden, are not consistently examined [28, 29, 34]. Although behavioral and supportive strategies, such as patient education, counseling, and social support during treatment initiation, have been suggested to facilitate continued medication use, descriptions of these approaches remain limited in the current literature for this dual condition population [14, 29, 35, 36].

The purpose of this narrative review is to summarize existing evidence on treatment persistence with GLP-1–based therapies among adults with obesity and type 2 diabetes. Specifically, this review examines how persistence has been defined and measured, describes real-world persistence outcomes, and summarizes factors associated with variation in treatment continuation and discontinuation. It also synthesizes reported reasons for discontinuation and explores the extent to which behavioral or supportive strategies are described in the literature.

## Methodology

### Study Design

This study was conducted as a narrative review. A structured approach was used to identify relevant studies, including development of a search strategy and application of predefined eligibility criteria. Findings were synthesized qualitatively to summarize patterns across studies. This review aimed to examine how treatment persistence is defined and reported in real-world studies, and to summarize patterns of treatment continuation, discontinuation, and associated factors in this population.

## Search Strategy

A structured literature search was conducted in PubMed, Scopus, and Embase to identify studies examining treatment persistence with GLP-1–based therapies among adults with obesity and type 2 diabetes. The search was limited to studies published from January 1, 2021 through May 2026, corresponding to the period when GLP-1 receptor agonists and dual GIP/GLP-1 receptor agonists became widely used for chronic weight management. The search strategy combined terms related to type 2 diabetes, obesity, GLP-1–based therapies, and treatment persistence. Terms included Medical Subject Headings, controlled vocabulary terms when available, and keywords such as “type 2 diabetes,” “obesity,” “GLP-1 receptor agonists,” selected agents such as semaglutide and tirzepatide, and persistence-related terms such as “persistence,” “adherence,” and “discontinuation.” Additional terms related to demographic and socioeconomic characteristics were included to capture variation across patient subgroups. Searches were restricted to English-language publications with available abstracts. The complete search terms and database-specific search strategies are provided in Online Resource 1 (Supplementary Table 1).

## Study Selection

Studies identified through the literature search were screened for relevance based on predefined eligibility criteria aligned with the objectives of this review. Duplicate records across databases were removed before screening. Titles and abstracts were screened first, followed by full-text review of potentially eligible studies. Studies were included if they examined adults with both obesity and type 2 diabetes and evaluated treatment persistence with GLP-1–based therapies, including GLP-1 receptor agonists or dual GIP/GLP-1 receptor agonists. Studies reporting a clearly identifiable subgroup with both obesity and type 2 diabetes were also eligible if the subgroup could be identified based on study inclusion criteria, diagnostic codes, body mass index (BMI) categories, medication indications, or baseline clinical characteristics. Included studies were required to report persistence in a way that allowed interpretation of treatment continuation or discontinuation over time, such as persistence rates, time to discontinuation, treatment duration, discontinuation risk, or refill-based measures. Studies were also required to provide information on variation across demographic, clinical, treatment-related, or methodological characteristics. Studies focused solely on treatment initiation, short-term outcomes, or populations without clear overlap of obesity and type 2 diabetes were excluded. Detailed criteria are provided in Online Resource 1 (Supplementary Table 2). The study selection process was documented using a flow diagram adapted from the PRISMA 2020 flow diagram template [37].

## Data Extraction

Data were extracted to summarize study characteristics, population context, and how treatment persistence was defined and measured. Reported persistence outcomes and factors associated with variation across demographic, clinical, treatment-related, or methodological characteristics were also recorded. When available, reasons for treatment discontinuation were noted, along with any descriptions of behavioral or supportive strategies related to continued treatment use. Findings were summarized narratively and organized in tables to highlight differences in persistence definitions, patterns across populations, and reported reasons for discontinuation.

### Data Analysis

Given variation in persistence definitions, follow-up periods, and study populations, findings were synthesized narratively rather than quantitatively pooled. The analysis focused on patterns in how persistence was defined and reported, observed persistence outcomes, factors associated with continuation or discontinuation, and the extent to which reasons for discontinuation and behavioral supportive strategies were described.

## Results

### Overview

**Fig. 1:**
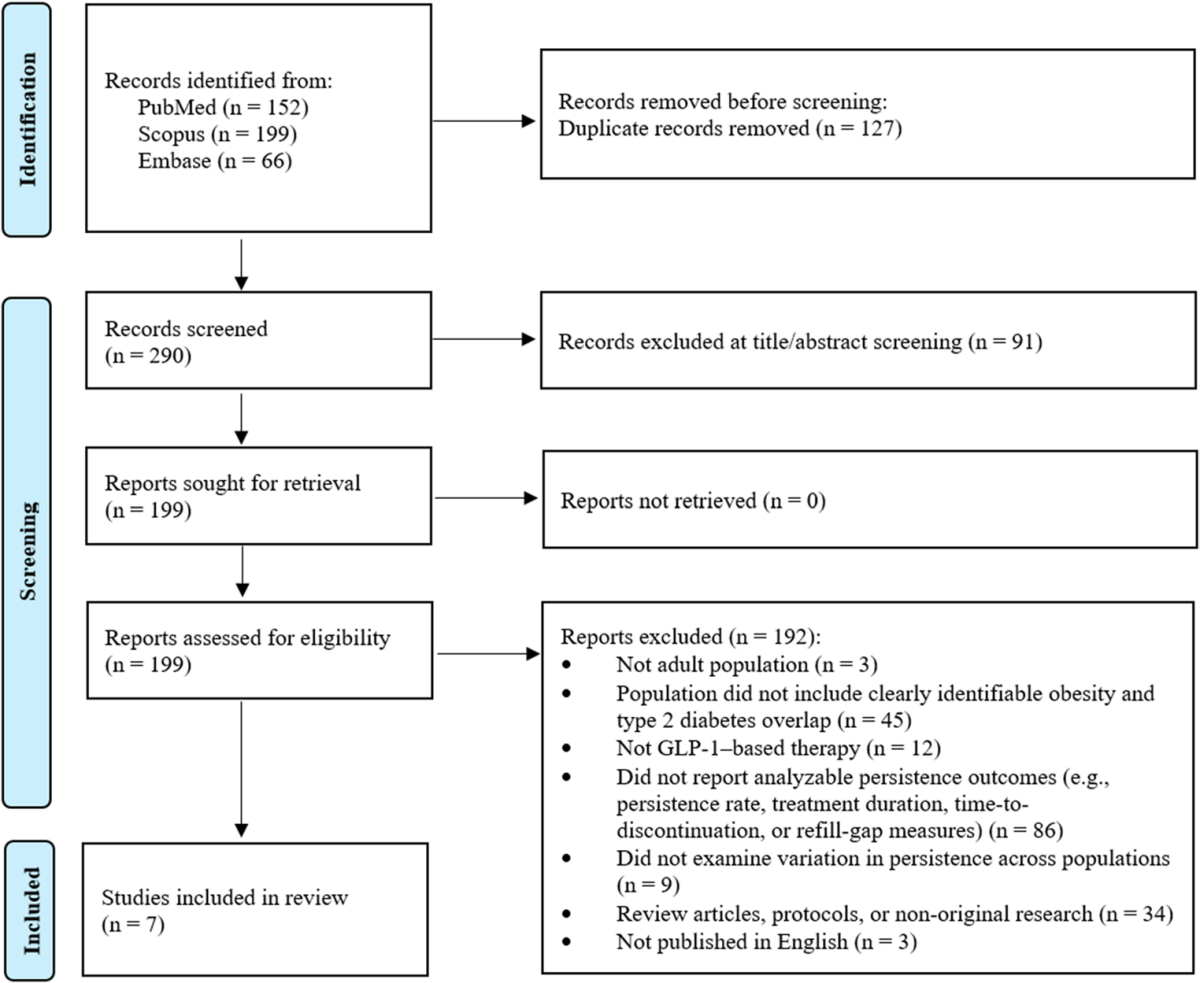
Flow diagram of the literature search and study selection process. Adapted from the PRISMA 2020 flow diagram [37] under the Creative Commons Attribution 4.0 International License; changes were made to reflect the search and study selection process for this narrative review

Database searches identified 417 records, including 152 from PubMed, 199 from Scopus, and 66 from Embase. After duplicate removal, 290 unique records were screened. Following title/abstract screening and full-text review, a total of 7 studies met the inclusion criteria and were included in the final narrative synthesis [28, 38–43]. The study selection process is summarized in Fig. 1. The included studies varied in sample size, population characteristics, study design, and follow-up duration, reflecting the heterogeneity in how persistence to GLP-1–based therapies has been examined across real-world settings.

## Study Characteristics and Definitions of Persistence

The seven included studies were published between 2021 and 2026 and were all retrospective real-world analyses using administrative claims or electronic health record data (Table 1). Although all studies focused on GLP-1–based therapies among adults with obesity and type 2 diabetes, there were clear differences in how treatment persistence was defined and measured [28, 38–43].

Most studies used a gap-based definition of persistence, with permissible gaps ranging from 30 to 90 days without medication refills or prescriptions. For example, Rodriguez et al. (2025) defined discontinuation as ≥ 60 consecutive days without any GLP-1 RA medication supply [28]. Ulrich et al. (2026) defined discontinuation as a > 90-day gap between consecutive GLP-1 RA prescriptions, with switching between GLP-1 RA agents not considered discontinuation in the primary analysis [43]. In contrast, Palanca et al. (2023) and Mirabelli et al. (2021) defined persistence based on the proportion of patients remaining on a GLP-1 therapy at specific time points [41, 42].

Follow-up periods ranged from 6 months to 2 years. Several studies reported 1-year persistence rates, while Rodriguez et al. used time-to-event analyses over 2 years to estimate discontinuation and reinitiation [28, 38–43]. These differences in definitions, analytic approaches (e.g., binary measures vs. time-to-event models), and follow-up periods make direct comparisons across studies difficult and support the use of a narrative summary. Furthermore, none of the studies measured persistence using patient self-report; instead, all of the studies relied on prescription refill or electronic health record data.

**Table 1:** Characteristics of included studies and definitions of treatment persistence

Study (Year, Country)	Study design / data source	GLP-1 agent(s)	Population context (BMI / T2D description)	Persistence definition	Follow-up duration
Ulrich et al. (2026, UK) [43]	Retrospective cohort study EHR data	Dulaglutide (46.5%) Semaglutide (38.7%) Liraglutide (11.9%)	• *N* = 4,963 (Obesity subgroup) • Adults with T2D initiating GLP-1 RA therapy • Obesity: BMI ≧ 30 kg/m² • Median BMI: 34.0 kg/m² • Age: 60.7 [53.1, 68.6]	Prescription gap < 90 days	12 months
Bowe et al. (2026, USA) [38]	Retrospective cohort study Medicare Advantage claims data	Dulaglutide (45.4%) Semaglutide (40.3%) Liraglutide (11.9%)	• *N* = 12,276 (Obesity subgroup) • Adults with T2D • Obesity: BMI ≧ 30 kg/m² or obesity diagnosis code • Age: 68.1 ± 8.4 ^a^	Days’ supply gap < 45 days	12 months
Rodriguez et al. (2025, USA) {28}	Retrospective cohort study Claims data	Liraglutide (13.0%) Semaglutide (74.1%) Tirzepatide (13.0%)	• *N* = 76,524 (T2D subgroup) • Adults initiating GLP-1 RA therapy • With T2D and obesity • Median BMI: 37 kg/m² • Age: 54.4 ± 13.1 ^a^	Days’ supply gap < 60 days	2 years
Gasoyan et al. (2024, USA) [39]	Retrospective cohort study EHR data	Semaglutide (48.1%) Liraglutide (51.9%)	• *N* = 2,785 (T2D subgroup) • Adults with T2D and Obesity • BMI ≧ 30 kg/m² • Age: 50.4 ± 12.2 ^a^	Cumulative days’ supply gap < 90 days	12 months
Lee et al. (2023,South Korea) [40]	Retrospective cohort study EHR data	Liraglutide (57.2%), Lixisenatide (42.8%)	• *N* = 166 (T2D subgroup) • Adults with T2D and Obesity • BMI ≧ 30 kg/m² • Age: Liraglutide: 47.2 ± 12.7 • Lixisenatide: 43.8 ± 12.1	Prescription gap < 1 month	6 months
Palanca et al. (2023, Spain) [42]	Retrospective cohort study EHR data	GLP-1 receptor agonists	• *N* = 1,848 (GLP-1 RA subgroup) • Adults with T2D and Obesity • BMI ≧ 30 kg/m² Age: 57.7 ± 10.7	Percentage of patients remaining on therapy at 1 and 2 years	2 years
Mirabelli et al. (2021, Italy) [41]	Retrospective cohort study EHR data	Dulaglutide	• *N* = 126 • Adults with T2D and overweight/obesity • Overweight: BMI ≧ 25 kg/m² • Obesity: BMI ≧ 30 kg/m² • Age: 59.8 ± 9.4	Percentage of patients remaining on therapy at 18 months	18 months

Abbreviations: *BMI *body mass index; *T2D *type 2 diabetes; *GLP-1 *glucagon-like peptide-1; *EHR *electronic health record

^a^Age reported for the overall study population rather than the specific obesity and type 2 diabetes subgroup

### Reported Persistence Outcomes

Persistence outcomes varied across studies and were strongly influenced by how persistence was defined and the length of follow-up (Table 2). Across the seven studies, persistence generally declined over time, with substantial discontinuation occurring within the first one to two years of therapy [28, 38–43].

Across cohorts with longer follow-up, persistence commonly fell below 60% by 12 to 24 months, indicating that a substantial proportion of patients did not remain on therapy over time [28, 38–43]. Estimates varied depending on methodological choices. For example, Bowe et al. (2026) showed that wider allowable refill gaps were associated with higher persistence estimates, with 12-month persistence ranging from 32.8% to 51.0% depending on the gap definition [38].

Patterns of treatment also suggested that persistence is not always continuous. Rodriguez et al. (2025) reported that discontinuation exceeded 60% by two years, and nearly half of patients reinitiated therapy within the first year, indicating frequent treatment interruption and cycling rather than permanent discontinuation [28].

### Factors Associated with Persistence and Discontinuation

Variation in persistence or discontinuation was observed across both patient and treatment-related factors (Table 2). Differences in persistence were observed across patient characteristics, including demographic, socioeconomic, and clinical factors. In Rodriguez et al. (2025), older age (≥ 65 years), lower income, and on-treatment moderate to severe gastrointestinal adverse events were associated with a higher risk of discontinuation, with discontinuation rates exceeding 60% at 2 years in this cohort. In contrast, greater weight reduction during therapy was associated with a lower risk of discontinuation, with each 1% decrease in body weight corresponding to a 3.1% reduction in the hazard of discontinuation [28]. Across studies, body weight and obesity-related measures appeared to be linked with continued treatment. In Mirabelli et al. (2021), higher baseline BMI was associated with a lower risk of discontinuation due to gastrointestinal adverse events, with an odds ratio of 0.21 (95% CI 0.06–0.77) [41]. Similarly, Ulrich et al. (2026) reported lower discontinuation among patients with BMI-defined obesity than among those with BMI < 30 kg/m² (40.4% and 37.2% vs. 49.2%), suggesting that patients with obesity may be more likely to remain on therapy in some settings [43].

Differences in persistence were also observed across treatment-related factors, including drug type and dosing schedule. Gasoyan et al. (2024) reported higher 1-year persistence among semaglutide users compared with liraglutide users (45.8% vs. 35.6%) [39]. Ulrich et al. (2026) also found variation by GLP-1 RA agent and formulation, with lower 1-year discontinuation among dulaglutide users than semaglutide or liraglutide users (36.9% vs. 48.7% and 53.8%) and higher discontinuation among oral semaglutide users than subcutaneous semaglutide users (55.8% vs. 46.4%) [43]. Palanca et al. (2023) also observed variation in persistence by dosing schedule, with lower persistence among weekly users compared with daily users (77.0% vs. 87.0%) [42]. Lee et al. (2023) similarly found differences in treatment duration across GLP-1 agents, with shorter persistence observed across agents overall [40]. However, these treatment-related patterns were not fully consistent across studies, likely reflecting differences in populations, type of GLP-1 agents, follow-up periods, and persistence definitions.

These findings suggest that treatment persistence varies across both patient characteristics and treatment-related factors in real-world settings, but the direction and magnitude of these associations depend on study context and measurement approach.

**Table 2:** Treatment persistence outcomes and factors associated with continuation or discontinuation

Study (Year)	Main persistence result	Variation in persistence across patient and methodological characteristics
Ulrich et al. (2026, UK) [43]	• 1-year discontinuation: - BMI 30 – < 35 kg/m²: 40.4% - BMI ≧ 35 kg/m²:37.2%	• BMI: Patients with BMI < 30 kg/m² had higher 1-year discontinuation than those with BMI 30–<35 or ≥ 35 kg/m² (49.2% vs. 40.4% and 37.2%) • Treatment: Dulaglutide had lower 1-year discontinuation than semaglutide or liraglutide (36.9% vs. 48.7% and 53.8%) • Formulation: Oral semaglutide had higher 1-year discontinuation than subcutaneous semaglutide (55.8% vs. 46.4%)
Bowe et al. (2026) [38]	• 12-month persistence (45-day gap): 38.9% • Persistence increased with wider gap definitions: – 30-day: 32.8% – 90-day: 51.0%	• Definition: Wider allowable gaps increased persistence estimates (30-day: 32.8%; 45-day: 38.9%; 90-day: 51.0%)
Rodriguez et al. (2025) [28]	• Discontinuation: – 1 year: 46.5% – 2 years: 64.1% • Reinitiation: – Within 1 year: 47.3% – Within 2 years: 57.3%	• Age: Older age (≥ 65 years) associated with higher discontinuation (HR 1.28, 95% CI 1.24–1.32) • Socioeconomic: Higher income associated with lower discontinuation (HR 0.72, 95%CI, 0.69–0.76) • Clinical: Greater weight loss associated with lower discontinuation (3.1% lower hazard per 1% reduction, 95% CI 2.9–3.2) • Adverse events: GI adverse events associated with higher discontinuation (HR 1.38, 95% CI 1.31–1.45)
Gasoyan et al. (2024) [39]	• 1-year persistence: – Semaglutide: 45.8% – Liraglutide: 35.6%	• Treatment: Semaglutide associated with higher persistence than liraglutide (*p* < 0.001)
Lee et al. (2023)[40]	• 6-month discontinuation: – Liraglutide: 73.7% – Lixisenatide: 63.4% • Persistence (treatment duration): – Liraglutide: 3.5 ± 0.2 months – Lixisenatide: 2.8 ± 0.3 months	• Treatment: Liraglutide associated with longer persistence than lixisenatide (*p* < 0.05)
Palanca et al. (2023) [42]	• Persistence: – 1 year: 81.5% – 2 years: 45.5%	• Treatment: Weekly users associated with lower persistence compared with daily users (1-year persistence 77.0% vs. 87.0%, *p* < 0.0001) • Clinical outcome: Persistence associated with higher likelihood of HbA1c reduction (40.6% vs. 18.6%, *p* < 0.0001)
Mirabelli et al. (2021) [41]	• 18-month persistence: 51.6% • Discontinuation due to GI events: 10.3%	• Clinical: Higher baseline BMI (≥ 30 kg/m²) associated with lower discontinuation (OR 0.21, 95% CI 0.06–0.77)

Abbreviations: *BMI *body mass index; *T2D *type 2 diabetes; *GLP-1 *glucagon-like peptide-1; *HR *hazard ratio; *OR *odds ratio; *CI *confidence interval; *GI *gastrointestinal; *HbA1c *hemoglobin A1c

### Reported Reasons for Discontinuation and Behavioral Strategies

Only two of the seven included studies explicitly reported patient-level reasons for treatment discontinuation [40, 41]. Across these studies, adverse effects were the most commonly reported reason for stopping therapy.

In Lee et al. (2023), most patients who discontinued treatment attributed this to side effects, including nausea, vomiting, diarrhea, dizziness, and headache. Among those discontinuing liraglutide and lixisenatide, 68.6% and 64.4% of patients, respectively, reported adverse effects were the primary reason. Discontinuation due to lack of perceived effectiveness or switching to another anti-obesity medication was also reported, accounting for 31.4% of discontinuations among liraglutide users and 35.5% among lixisenatide users [40]. Similarly, Mirabelli et al. (2021) reported that 10.3% of patients discontinued dulaglutide due to moderate-to-severe gastrointestinal adverse events [41].

Most of the remaining studies did not report specific patient-level reasons for discontinuation. In addition, none of the included studies described structured behavioral or supportive strategies intended to promote continued use of GLP-1–based therapies. This limited reporting of both discontinuation mechanisms and behavioral or supportive strategies highlights an important gap in the current literature and limits understanding of factors influencing long-term treatment continuation in real-world practice.

## Discussion

Persistence with GLP-1–based therapies in real-world settings reflects a dynamic process shaped by patient experiences and treatment-related factors. Although these therapies are intended for long-term use in the management of adults with obesity and type 2 diabetes, sustained use in routine clinical practice remains difficult [7, 28, 38–43]. One explanation relates to the timing of treatment burden relative to perceived benefit. Gastrointestinal adverse effects often occur early in treatment, particularly during dose escalation, whereas clinically meaningful benefits such as weight reduction may take longer to emerge [40, 41]. This difference in timing may contribute to early discontinuation before patients experience the full therapeutic effect. Practical and structural factors also appear to influence persistence. Medication cost, access, and the burden of ongoing injectable therapy may limit sustained use in real-world settings [28, 40]. Treatment often occurs in environments with limited structured behavioral support or ongoing counseling, which may reduce patients’ ability to manage side effects and remain engaged over time [13, 14]. Persistence can therefore be understood as reflecting an ongoing balance between treatment tolerability, perceived benefit, and broader contextual factors that shape long-term use.

Prior reviews have largely focused on the clinical benefits and safety of GLP-1–based therapies, with less attention to how treatment persistence is defined and measured in real-world settings [44–46]. In the studies included in this review, persistence was operationalized using different approaches, most commonly based on allowable refill gaps, prescription gaps, or the proportion of patients remaining on therapy at specific time points [28, 38–43]. These approaches do not directly observe treatment use but rely on study-specific definitions, meaning that reported estimates are sensitive to how persistence is constructed. Even modest differences in allowable gaps can lead to substantial variation in reported persistence rates, complicating interpretation across studies [38]. Most studies relied on prescription claims or electronic health record data, which capture medication prescribing or dispensing rather than actual use [28, 38–43]. As a result, these measures provide limited insight into treatment interruptions, reinitiation, or the reasons underlying discontinuation. Persistence is often treated as a binary outcome, yet real-world patterns appear more dynamic, with some patients discontinuing and later restarting therapy [20, 28]. This suggests that persistence may be better understood as a pattern of treatment use over time rather than a single endpoint [20]. More consistent definitions and reporting approaches would improve comparability and support clearer interpretation of persistence across studies.

Differences in treatment persistence across studies appear to reflect the combined influence of demographic characteristics, patient experiences, and treatment-related factors. Older age and the occurrence of gastrointestinal adverse events were associated with a higher likelihood of discontinuation, whereas greater weight reduction during therapy was associated with improved continuation [28]. These findings suggest that persistence is closely tied to patients’ experiences during treatment, particularly the balance between treatment tolerability and perceived benefit. Gastrointestinal symptoms commonly occur during dose escalation and may discourage continued use, especially in the early phase of treatment when clinical benefits such as weight reduction may not yet be fully realized [40, 41]. In contrast, patients who experience meaningful weight loss or metabolic improvement may perceive greater value in continuing therapy despite potential side effects [28]. Obesity-related characteristics may also influence this balance. Patients with clearer weight-related treatment goals may be more motivated to continue therapy if they perceive early benefits or expect meaningful weight reduction over time [41, 43].

In addition to these patient-level experiences, characteristics of the treatment regimen also appear to influence persistence. However, treatment-related patterns were not uniform across studies. Differences by drug type, dosing schedule, and formulation suggest that persistence may depend not only on dosing frequency, but also on tolerability, route of administration, clinical indication, and how well treatment fits into patients’ routines [39, 40, 42, 43]. Similar patterns have been observed in the broader medication literature, where regimens requiring fewer dosing events are often associated with improved long-term use, likely due to reduced day-to-day treatment burden and easier integration into daily routines [47, 48]. In the GLP-1 literature, however, convenience alone may not fully explain persistence, because agent- and formulation-specific differences may reflect multiple factors, including side-effect profiles, patient preference, access, treatment expectations, and prescribing context [28, 40–43].

Treatment persistence was not a primary focus in several of the included studies and was typically reported as one component of broader evaluations of weight reduction, glycemic control, or overall treatment effectiveness [28, 38–43]. As a result, discontinuation was often identified without detailed examination of the underlying reasons. Only a limited number of studies reported patient-level factors associated with stopping therapy, most commonly gastrointestinal adverse events [40, 41]. Other potentially relevant factors, including expectations of treatment benefit, financial barriers, and challenges related to long-term injectable therapy, were infrequently explored [29, 34]. This suggests that current evidence captures when treatment is discontinued but provides limited insight into why discontinuation occurs. Consequently, the mechanisms underlying treatment discontinuation remain insufficiently characterized, which constrains the development of targeted strategies to support long-term treatment continuation.

Structured behavioral or supportive strategies to promote long-term continuation were largely absent from the included studies [28, 38–43]. Polonsky and Henry (2016) highlighted the importance of interventions such as patient education, counseling on side effects, and ongoing provider support in helping patients remain on therapy and reducing discontinuation. However, none of the included studies described or evaluated such strategies, suggesting an important gap in understanding how persistence with GLP-1–based therapies may be supported in real-world settings [29]. Several studies acknowledged the need for additional support to sustain treatment over time, particularly in managing adverse effects and maintaining patient engagement [39, 40, 42, 43]. Sustained use of GLP-1–based therapies reflects both pharmacologic effects and how treatment is introduced and supported in clinical care [29, 35]. Clinicians may provide education about expected adverse effects, guidance during dose escalation, and counseling on realistic weight trajectories to help patients navigate early treatment challenges [36]. These approaches are also reflected in current diabetes care guidelines, which emphasize patient education and ongoing support as part of long-term treatment management [14]. Yet such support was rarely described, even in studies reporting substantial treatment discontinuation. Overall, these observations point to persistence as being shaped not only by individual patient experiences, but also by the extent to which structured support is incorporated into clinical care [49]. As a result, the existing literature offers limited insight into how persistence might be improved through practical interventions in care settings.

Overall, these findings suggest that persistence with GLP-1–based therapies in real-world settings is dynamic rather than continuous, and may involve periods of interruption, discontinuation, and reinitiation over time. This pattern highlights that treatment continuation is not solely determined at initiation but evolves as patients reassess the balance between treatment benefits and burdens throughout the course of therapy. From a clinical and research perspective, these observations underscore the importance of moving beyond binary definitions of persistence toward a more nuanced understanding of treatment trajectories. Approaches that capture patterns of use over time may better reflect real-world treatment behavior and provide more meaningful insight into long-term management. At the same time, the current evidence base remains limited, particularly among individuals with both obesity and type 2 diabetes. Further research that explicitly examines persistence as a primary outcome and evaluates strategies to support sustained treatment use may help clarify how long-term continuation can be improved in routine clinical settings.

### Limitations

Several limitations should be considered when interpreting these findings. The number of eligible studies was relatively small, reflecting the limited evidence specifically examining persistence with GLP-1–based therapies among adults with both obesity and type 2 diabetes. Definitions of persistence also varied across studies, including differences in allowable refill gaps, analytic approaches, and follow-up duration, which limited direct comparison and made it difficult to distinguish true differences in treatment use from methodological variation. In addition, few studies were specifically designed for this dual-condition population; in many cases, these patients were identified through subgroup analyses within broader cohorts, which may limit how well the findings reflect this population as a distinct clinical group. Most studies relied on administrative claims or electronic health record data, which capture prescription or dispensing information rather than actual medication use, treatment intent, or detailed insight into patient experiences and reasons for discontinuation. Finally, because the search strategy emphasized persistence and pharmacologic treatment terms, studies describing behavioral or supportive interventions may have been under identified.

## Conclusion

Persistence with GLP-1–based therapies among adults with obesity and type 2 diabetes appears to be variable in real-world clinical settings, with many patients discontinuing treatment within the first one to two years. Reported estimates differed across studies, likely reflecting variation in persistence definitions and study design. In addition, relatively few studies have specifically focused on individuals with both obesity and type 2 diabetes, despite the growing clinical relevance of this population. Although several patient- and treatment-related characteristics have been associated with variation in persistence, the underlying processes of treatment discontinuation remain only partially understood. Further research that explicitly examines persistence and explores strategies to support sustained treatment use may provide additional insight into long-term continuation in routine clinical practice.

## Supplementary information

Below is the link to the electronic supplementary material.

Supplementary Material 1

## Data Availability

No datasets were generated or analysed during the current study.

## Key references

- Rodriguez PJ, Zhang V, Gratzl S, Do D, Goodwin Cartwright B, Baker C, et al. Discontinuation and Reinitiation of Dual-Labeled GLP-1 Receptor Agonists Among US Adults With Overweight or Obesity. JAMA Netw Open. 2025;8(1):e2457349. 
  - ○ This large cohort demonstrates that discontinuation and reinitiation are common within two years of treatment initiation, supporting the view of persistence as a dynamic process rather than a single 
- Ceriello A, Prattichizzo F, Mastan Sheik Abdullah AR, La Grotta R, Berra CC, McGowan B, et al. Causes and consequences of discontinuation of GLP1RAs or tirzepatide. Nat Rev Endocrinol. 2026.
  - ○ This review synthesizes the causes and cardiometabolic consequences of discontinuing GLP-1 receptor agonists or tirzepatide and highlights the potential risks associated with repeated cycling on and off therapy.
- Ulrich FS, Napoli N, Nielsen MF, Burden AM. Real-world persistence and dose titration of GLP-1 receptor agonists in type 2 diabetes: A UK population-based cohort study by obesity and cardiovascular disease status. Diabetes Obes Metab. 2026;28(4):3386–95.
  - ○ This population-based cohort demonstrates that discontinuation varies by GLP-1 receptor agonist, formulation, and obesity status, showing that persistence reflects both treatment- and patient-level factors.
- Bowe A, Hayes M, John I, Diaz M, Dixon S, Poonawalla I. Glucagon-like peptide-1 receptor agonist versus sodium-glucose cotransporter-2 inhibitor persistence in Medicare Advantage beneficiaries with type 2 diabetes, cardiovascular risk, and obesity. Diabetes Obes Metab. 2026;28(1):472–84.
  - ○ This study demonstrates that 12-month persistence estimates vary substantially according to the allowable refill-gap definition, highlighting the need for more standardized persistence measures.
