# Supplementary material for: Persistence in GLP-1–Based Therapy Among Adults with Obesity and Type 2 Diabetes: A Narrative Review of Definitions, Real-World Outcomes, and Determinants
Source: Curr Diab Rep. 2026 Aug 15;26(1):25. doi: 10.1007/s11892-026-01639-0 (PMC13477449; doi:10.1007/s11892-026-01639-0)
Supplement: Supplementary file 1 [file 11892_2026_1639_MOESM1_ESM.docx]

**Online Resource 1.**

Supplementary Table 1. Searching process on PubMed and Scopus

Search Term for PubMed

- ("Diabetes Mellitus, Type 2"[MeSH] OR "type 2 diabetes" OR T2D) AND ("Obesity"[MeSH] OR obesity OR "weight management") AND ("Glucagon-Like Peptide 1 Receptor Agonists"[MeSH] OR "GLP-1" OR "dual GIP/GLP-1" OR semaglutide OR tirzepatide OR liraglutide OR dulaglutide OR exenatide OR lixisenatide) AND (persistence OR adherence OR discontinuation OR continuation) AND (age OR sex OR gender OR race OR ethnicity OR income OR socioeconomic OR insurance OR rural OR urban OR geographic OR "healthcare access")
- Filters: Abstract, Publication date from January 1, 2021 to May 13, 2026 / Sort by: Best Match
- Identified 152 results on May 13, 2026

Search Term for Scopus

- TITLE-ABS-KEY ( ( "type 2 diabetes" OR "type 2 diabetes mellitus" OR T2D ) AND ( obes* OR overweight OR "weight management" OR BMI ) AND ( "glucagon-like peptide 1 receptor agonist*" OR "GLP-1" OR GLP1 OR "GLP-1 RA*" OR "GLP-1RA*" OR "dual GIP/GLP-1" OR "GIP/GLP-1" OR semaglutide OR tirzepatide OR liraglutide OR dulaglutide OR exenatide OR lixisenatide ) AND ( persist* OR discontinu* OR "treatment continuation" OR "treatment discontinuation" OR "treatment duration" OR "time to discontinuation" OR "refill gap*" OR "prescription gap*" ) AND ( age OR sex OR gender OR race OR ethnic* OR income OR socioeconomic OR insurance OR rural OR urban OR geographic* OR "healthcare access" OR "health care access" OR disparit* ) ) AND PUBYEAR AFT 2020 AND PUBYEAR BEF 2027
- Identified 199 results on May 13, 2026

Search Term for Embase

- ('type 2 diabetes':ti,ab,kw OR 'type 2 diabetes mellitus':ti,ab,kw OR t2d:ti,ab,kw) AND (obes*:ti,ab,kw OR overweight:ti,ab,kw OR 'weight management':ti,ab,kw) AND ('glucagon-like peptide 1 receptor agonist':ti,ab,kw OR 'glucagon-like peptide 1 receptor agonists':ti,ab,kw OR 'glucagon like peptide 1 receptor agonist':ti,ab,kw OR 'glucagon like peptide 1 receptor agonists':ti,ab,kw OR 'glp-1':ti,ab,kw OR glp1:ti,ab,kw OR 'glp-1 ra':ti,ab,kw OR 'glp-1 receptor agonist':ti,ab,kw OR 'glp-1 receptor agonists':ti,ab,kw OR 'dual gip/glp-1':ti,ab,kw OR 'gip/glp-1':ti,ab,kw OR semaglutide:ti,ab,kw OR tirzepatide:ti,ab,kw OR liraglutide:ti,ab,kw OR dulaglutide:ti,ab,kw OR exenatide:ti,ab,kw OR lixisenatide:ti,ab,kw) AND (persist*:ti,ab,kw OR discontinu*:ti,ab,kw OR nonpersist*:ti,ab,kw OR 'non-persist*':ti,ab,kw OR 'treatment continuation':ti,ab,kw OR 'treatment discontinuation':ti,ab,kw OR 'treatment duration':ti,ab,kw OR 'time to discontinuation':ti,ab,kw OR 'medication gap':ti,ab,kw OR 'refill gap':ti,ab,kw OR 'prescription gap':ti,ab,kw) AND (age:ti,ab,kw OR sex:ti,ab,kw OR gender:ti,ab,kw OR race:ti,ab,kw OR ethnic*:ti,ab,kw OR income:ti,ab,kw OR socioeconomic:ti,ab,kw OR 'socio-economic':ti,ab,kw OR insurance:ti,ab,kw OR rural:ti,ab,kw OR urban:ti,ab,kw OR geographic*:ti,ab,kw OR 'healthcare access':ti,ab,kw OR 'health care access':ti,ab,kw OR disparit*:ti,ab,kw) AND [2021-2026]/py AND [english]/lim AND [abstracts]/lim AND ('article'/it OR 'article in press'/it)
- Identified 66 results on May 16, 2026

Supplementary Table 2. Inclusion and exclusion criteria for study selection

Inclusion criteria

- Studies involving adults aged 18 years or older with both obesity and type 2 diabetes, or studies reporting a clearly identifiable subgroup with both conditions based on study inclusion criteria, diagnostic codes, BMI categories, medication indications, or baseline clinical characteristics.
- Studies examining GLP-1–based therapies, including GLP-1 receptor agonists or dual GIP/GLP-1 receptor agonists.
- Studies with a minimum follow-up duration of at least 3 months to allow meaningful assessment of treatment persistence.
- Studies reporting treatment persistence or discontinuation as an analyzable outcome (e.g., persistence rate, treatment duration, time-to-discontinuation, refill-gap measures).
- Studies providing information allowing evaluation of variation in persistence or discontinuation across demographic, clinical, or methodological characteristics (e.g., age, sex, socioeconomic factors, baseline clinical characteristics, drug types, or persistence definitions).
- Peer-reviewed articles published in English from January 2021 onward

Exclusion criteria

- Studies involving only type 1 diabetes, gestational diabetes, or pediatric populations.
- Studies focused solely on treatment initiation, short-term efficacy, or clinical outcomes without reporting persistence or discontinuation measures.
- Studies without clearly identifiable overlap between obesity and type 2 diabetes populations.
- Review articles, editorials, letters, protocols, conference abstracts without a full peer-reviewed report, or non-original research reports.
- Studies without sufficient information to interpret variation in treatment persistence across populations.
